# Community mobilization and household level waste management for dengue vector control in Gampaha district of Sri Lanka; an intervention study

**DOI:** 10.1179/2047773212Y.0000000060

**Published:** 2012-12

**Authors:** W Abeyewickreme, A R Wickremasinghe, K Karunatilake, Johannes Sommerfeld, Axel Kroeger

**Affiliations:** 1 University of Kelaniya, Faculty of Medicine, Thalagolla Road, Ragama, Sri Lanka; 2 University of Kelaniya, Faculty of Social Sciences, Dalugama, Kelaniya, Sri Lanka; 3 Special Programme for Research and Training in Tropical Diseases (TDR/WHO) Via Appia, Geneva, Switzerland; 4 Liverpool School of Tropical Medicine, Pembroke Place, Liverpoool, UK

**Keywords:** Targeted interventions, community participation, dengue vector management, *Aedes aegypti* control, Sri Lanka

## Abstract

**Introduction:**

Waste management through community mobilization to reduce breeding places at household level could be an effective and sustainable dengue vector control strategy in areas where vector breeding takes place in small discarded water containers. The objective of this study was to assess the validity of this assumption.

**Methods:**

An intervention study was conducted from February 2009 to February 2010 in the populous Gampaha District of Sri Lanka. Eight neighborhoods (clusters) with roughly 200 houses each were selected randomly from high and low dengue endemic areas; 4 of them were allocated to the intervention arm (2 in the high and 2 in the low endemicity areas) and in the same way 4 clusters to the control arm. A baseline household survey was conducted and entomological and sociological surveys were carried out simultaneously at baseline, at 3 months, at 9 months and at 15 months after the start of the intervention. The intervention programme in the treatment clusters consisted of building partnerships of local stakeholders, waste management at household level, the promotion of composting biodegradable household waste, raising awareness on the importance of solid waste management in dengue control and improving garbage collection with the assistance of local government authorities.

**Results:**

The intervention and control clusters were very similar and there were no significant differences in pupal and larval indices of *Aedes* mosquitoes. The establishment of partnerships among local authorities was well accepted and sustainable; the involvement of communities and households was successful. Waste management with the elimination of the most productive water container types (bowls, tins, bottles) led to a significant reduction of pupal indices as a proxy for adult vector densities.

**Conclusion:**

The coordination of local authorities along with increased household responsibility for targeted vector interventions (in our case solid waste management due to the type of preferred vector breeding places) is vital for effective and sustained dengue control.

## Introduction

Dengue infection has further spread in already endemic areas and to new areas in Asia since 2000.^12^ In 2003, Sri Lanka was one of eight Asian countries along with Bangladesh, India, Indonesia, Maldives, Thailand, Myanmar and Timor-Leste where outbreaks of dengue occurred. Since then, Sri Lanka has experienced severe outbreaks in 2007 and 2010 onwards. At present, epidemic dengue is a major public health problem in Sri Lanka situated in the tropical monsoon and equatorial zone.^18^

While *Aedes aegypti* is the major vector of dengue in Sri Lanka, *Aedes albopiuctus* is also widespread in both urban and rural areas where multiple serotypes are circulating. During the past decade, cyclic epidemics have increased in frequency with geographic extension of transmission within the country. The disease has become a major cause of death particularly among children^7^ with a case fatality rate of about 1% in 2010. Several possible reasons for the increase in dengue deaths have been postulated; these include shifting serotypes, introduction of new serotypes into the country,^7^ mutation of existing serotypes to more virulent forms, adaptation of vector species to more virulent virus forms and improper patient management. Whatever the reason, the increase in the number of dengue cases and deaths in the country, particularly during the past few years, has been alarming.

*Aedes aegypti,* a major vector of dengue transmission, mainly breeds in domestic environments.^2^ Its preferred habitats are water storage tanks and jars inside and outside houses, roof gutters, leaf axils, bamboo stumps and temporary containers such as drums, used car tyres, tin cans, bottles and plant pots. All these habitats typically contain relatively clean water. *Aedes albopictus,* originally seen only in Asia and Madagascar, has recently invaded North and South America, and West Africa, where it may become important in the transmission of dengue and other viral diseases. Like *Aedes aegypti*, it breeds in temporary containers but prefers natural ones in forests, such as tree holes, leaf axils, ground pools and coconut shells; it breeds more often outdoors in gardens and less frequently indoors in artificial containers.^15^ *Aedes* mosquitoes bite mainly in the morning or evening. Most species bite and rest outdoors but in tropical towns *Aedes aegypti* breeds, feeds and rests in and around houses.

Dengue was first reported in Sri Lanka in 1965 and, since 1989, it has demonstrated a regular seasonal pattern sometimes assuming epidemic proportions.^17^ The disease incidence peaks generally after the monsoon season, when the density of the two mosquito carrier species, *Aedes aegypti* and *Aedes albopictus* is especially high.^17^

The incidence of dengue is positively correlated with education or literacy level. The more educated the community is the less the incidence of the disease, probably due to increased awareness and ability to afford and practice prevention methods and strategies. Some studies have found that though communities may perform well in terms of knowledge of the disease, they may have poorer attitudes and fewer preventive practices. Therefore, behavioral change is an area to focus on in social mobilization programmes.^5^

The reduction of dengue transmission by elimination of breeding places of the vector mosquitoes, *Ae. aegypti* and *Ae*. *albopictus,* is the mainstay of dengue control. In the first phase of this study, it was observed that the most frequent container type (of all container types) of *Aedes* pupae was discarded containers (43.3%); 80.2% of *Aedes* pupae (a proxy measure for adult vectors) were present in discarded containers.^11^ As discarded containers play a major role serving as breeding habitats for *Aedes* mosquitoes, we conducted an intervention study in the Gampaha district of Sri Lanka focusing on proper solid waste management with community mobilization through awareness programmes and by introducing household level composting systems for biodegradable waste together with the introduction of improved garbage collecting systems for other non-biodegradable waste as a control strategy for dengue transmission. We report here on the process of the intervention, including its acceptance both by the provider and the community and its final outcome, the reduction of dengue vector density estimated by pupal indices as suggested by Focks^3^ and Focks and Alexander.^4^

## Study site and Methods

### Study period and setting

This study is part of the multi-country study on community centered ecosystem management for dengue vector control in six south-east Asian countries. The intervention study in Sri Lanka was a prospective experimental study carried out over a 12-month period from February 2009 to February 2010 in the Gampaha district of the Western Province of Sri Lanka comprising middle sized provincial towns, and peri-urban and rural areas. The Gampaha district is the second most populous district in the country and is situated adjacent to the Colombo district in which both the administrative and commercial capitals of the country lie. In 2008, the estimated district population was approximately 2.2 million. The district extends over 1,387 sq. kms and has a population density of approximately 1,800 persons per sq. km. In 2004/2005, paddy was cultivated in 10,170 hectares of the district. The second highest number of dengue cases has been reported from the Gampaha district over the last five years.

### Study design

The intervention study was conducted in four intervention and four control clusters each comprising 200 households.

### Sample size and selection

In order to detect a difference of 0.25 in the proportion of households with pupae between intervention and control clusters, with an alpha error of 5% and a power of 90%, four clusters each of 200 households is required in each arm assuming that the intraclass correlation coefficient is 0.01.

### Selection of study clusters and households

Four urban/semi urban intervention clusters and four urban/semi urban control clusters were randomly selected for the study from among the ten high and ten low endemic clusters selected for Phase I of the study (Arunachalam et al. 2010). Of the four selected clusters from each type of transmission pattern, two were randomly assigned to the treatment and two to the control arms of the study. There were a total of eight clusters comprising two high and two low transmission clusters in the treatment arm and two high and two low transmission clusters in the control arm of the study, including roughly 800 household in each arm of the study.

For Phase I of the study, the random selection of clusters was done by overlaying a grid on a Google Earth satellite map and selecting ten grids randomly from each of high and low transmission areas. High and low transmission areas were categorized by taking the median of the reported incidence of dengue cases between year 2000 and 2006 as the cutoff point. When selecting a cluster, consideration was given to the inclusion of different ecotypes (industrial, urban settlement, semi-urban vegetation, etc) for both control and intervention groups. From each selected grid, a road was randomly selected. Then a house number was randomly selected and that particular house was considered the central point of the cluster. The closest house from that house was then selected and the process was continued concentrically until the required 200 houses were selected. Each household was labeled with a sticker having a unique identification number for project follow-up activities.

### Research methods

#### Household surveys

A baseline household survey was conducted in 1,585 households from April-May, 2009 in which socio-demographic information was obtained by administering a questionnaire through trained interviewers. Heads of households whose ages ranged from 20–90 years comprising 1,312 (82.7%) males and 273 (17.3%) females were interviewed. Prior to administering the questionnaire, the details of the study including the objectives and the methodology were explained to the head of the household and informed written consent was obtained.

#### Entomological and sociological surveys at baseline and follow up

A team of eight well-trained entomological assistants conducted the entomological surveys under the supervision of the investigators. The team consisted of Senior Entomological Assistants of the Medical Research Institute of the Ministry of Health. The team conducted inspections on larval and pupal breeding sites in all households in intervention and control areas. The baseline entomological survey (round 1) was conducted in all eight clusters from April-May 2009 and the follow-up surveys were conducted in August-September, 2009, February-March, 2010, and August-September, 2010. During the surveys, all types of water holding containers were inspected for the presence of *Aedes* larvae/pupae and the number and type of container(s) positive for *Aedes* larvae or pupae were recorded as well as the number of pupae in each positive container. Presence of *Aedes* larvae in natural breeding habitats was also recorded. While larval indices were used to quantify the presence or absence of vectors in each cluster, the pupal counts were taken as a proxy for adult vector abundance.

### Measurement of effect of intervention

#### Assessment of community mobilization

Community mobilization was assessed through focus group discussions, key informant interviews and views of heads of households on the effects of waste management in dengue control during surveys done in November-December 2009, July-August 2010 and December 2010 in all intervention clusters and in November-December 2009, and December 2010 in the control clusters. Trained interviewers were used for this purpose. The final assessment was conducted by an independent research team comprising university undergraduates and graduates having a sociology background. All assessment tools and questionnaires were checked for completeness before data were entered into computer databases.

#### In-depth Key informant Interviews (KIIs)

Several KIIs, including key stakeholders, were conducted at the planning stage of the intervention as well as during the monitoring stage of project programmes and activities. The interviews were conducted using interview checklists.

#### Focus Group Discussions (FGDs)

22 FGDs were conducted with community representatives and selected volunteers in the four intervention clusters. These FGDs were conducted in the planning and in the monitoring stages. First, the findings of the situation analysis (phase I of the study) were discussed with community representatives of the four selected intervention clusters. Later on, a series of FGDs were conducted to assess the effectiveness of the intervention in Phase II of the study using a checklist.

Aspects of the community mobilization process, its impact to the community, stakeholders’ commitment and contribution, the identification of gaps that needed to be improved and overall outcome of the project were discussed using the checklists prepared for KIIs and FGDs. An independent moderator was used in the final series of KIIs and FGDs. Information was transcribed into written records by community mobilisers who were closely monitored by the sociologist of the project.

#### Gender Analysis

Data collected through KIIs and FGDs were used to perform gender analysis. Gender analysis was conducted in both phases (I and II) of the project.

#### Stakeholder Analysis

The project identified the primary and secondary stakeholders prior to the intervention. As illustrated in Figure 1, all stakeholders directed by broad arrows were considered primary stakeholders and those directed by thin lines were considered as secondary stakeholders. An analysis of all stakeholders was conducted identifying the role of each, their capacities, limitations in their services, gaps in the process of monitoring, and capabilities to reduce dengue transmission through a well-managed SWM system. The process of changes in knowledge, attitudes and practices focused on all primary stakeholders. The services essential to introduce a well-managed SWM system is directly related to secondary stakeholders. The research team located at the Faculty of Medicine, University of Kelaniya coordinated all work and interactions with primary and secondary stakeholders.

**Figure 1:**
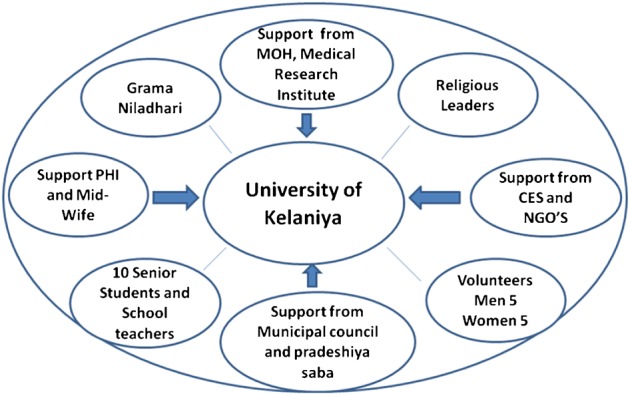
Primary and Secondary Stakeholders of the Project.

#### Overview of community interventions

The design of the intervention targeted water container types which were the most productive for vector breeding established through pupal surveys as described above. In the Gampaha district, small discarded containers such as bowls, tins and bottles were the most productive producing 80.2% of all *Aedes* pupae.^11^ Therefore, the intervention was aimed at improving solid waste management practices with community participation coupled with behavioral change in regard to maintaining a healthy environment. The following items were made available to households free of charge:

1. 150 litre compost bins.
2. Three bags for separation of solid waste (plastic, glass and paper).
3. Different varieties of vegetable and fruit plants for home gardening projects.

The compost bin was made of plastic and the garbage bags of polythene; they were purchased from the Central Environmental Authority (CEA) of Sri Lanka which is under the purview of the Ministry of Environment through local government bodies. A discount of 50% was given by the CEA for the compost bins of the project when purchased via the local government agency. There is a system for garbage collection through local government authorities but the system does not function regularly and effectively. The Central Environment Authority is promoting garbage separation throughout the country. Collected garbage is deposited in identified sanitary landfills.

#### Interventions for achieving behavioural change: Awareness programmes

All stakeholders including policy makers, local government authorities, religious and local leaders, public health officials and the general public were identified and informed about the project. The results of the baseline survey were presented and the objectives and methods of the intervention study were explained and discussed in detail.

Heads of households of intervention areas were requested to come to a pre-determined place for the distribution of compost bins and garbage disposal bags. During the distribution of compost bins and garbage disposal bags, generally done under patronage of a religious and/or a political leader, a programme (lecture/demonstration) was conducted by an expert on solid waste management to raise the awareness of the public regarding the importance of solid waste management, the principle of composting, the proper use of the bin and the advantages of composting. A brief presentation on home gardening and organic farming was also done. Three-to-four months after the distribution of compost bins, households were given plants for home gardening projects that would utilize their homemade compost fertilizer.

#### Coordination with Local Government Bodies

At the inception of the project, the investigators established close links with local government agencies and the public health officials of the area. In addition to sensitizing all these personnel on the problem of dengue, a situation analysis was done to identify strengths and weaknesses of the system in solid waste management and dengue control. Based on available logistics and resources at the local authority level, the support and cooperation of the local authority towards the project was assured, particularly in relation to raising community awareness and supporting garbage collection services.

#### Introduction and promotion of proper solid waste management at household level

At the initial field visit, the primary and secondary stakeholders and their capacities were identified. As a second step, the team conducted a series of Key Informant Interviews (KIIs) and Focus Group Discussions (FGDs) with these stakeholders to confirm their capacity and intention to support project activities.

In the process of identifying and communicating with the stakeholders, a group of volunteers comprising ten members representing male and female heads of households was selected from each intervention cluster. They were given a thorough understanding about the project and the project activities, and their active involvement in the project was solicited.

#### Volunteers

From the onset of the project, community volunteers participated in a one-day awareness raising programme conducted by the investigators at the Faculty of Medicine, University of Kelaniya. They participated in a subsequent follow-up programme as well. During the project, the volunteers organized many community activities and were the liaison between the community and the project staff. A close collaboration between volunteers and local government authorities was established with a long-term view for the sustainability of activities when funding of the project ceases. In the later stages of the project, volunteers were encouraged to form their own ‘Environmental and Health Associations’ for each locality to ensure the sustainability of project activities.

#### Involvement of school children and cleaning campaigns

An awareness programme for school children was conducted in eight schools in all intervention clusters. All senior students (Grade 10 and above), teachers and the principals of the schools actively participated in the awareness programme.

Several cleaning campaigns of the environment were organized by volunteer groups in their respective clusters with the active participation of local authorities, health workers (PHI and MOH), and religious leaders of the area.

#### Mobilization of Community towards Project Goals

The target communities were selected from one or two of the smallest administrative division/s, known as Grama Niladhari Division(s), of the district. After selection of these clusters, more attention was paid to the intervention clusters by identifying all relevant stakeholders of the planned activities. All stakeholders including school children were given an active role in the project.

With mobilization of the community, monthly monitoring and quarterly assessments were conducted in each cluster to identify the progress of project activities.

These assessments were conducted using checklists and questionnaires. The questionnaires contained both quantitative and qualitative assessments. Finally, a series of KIIs and FGDs were conducted to gather qualitative data focusing on project activities and its sustainability.

#### Intervention methods in control clusters

In the control clusters, routine surveillance activities were conducted in parallel to those conducted in the intervention clusters. There was no specific intervention at household level regarding waste separation and composting. However, public utilities and services that are routinely available through local government agencies were available to these clusters. Access to educational material and routine services that may impact on vector densities were equally available in control and intervention clusters.

#### Data analysis

In-depth qualitative and quantitative analyses were carried out. The number of pupae per person (PPP) was calculated as the total number of pupae found divided by the total number of persons per cluster and then multiplied by 100. The mean % reduction in pupae per person over the clusters per study arm was calculated as the mean of the percent reduction in the number of pupae per person in each round relative to the baseline value. Comparisons were done using Student’s t-tests.

A negative binomial model was used to model the number of pupae at household level adjusting for confounding variables that were significantly different at baseline between the intervention and control arms and the baseline number of pupae. The education status of the head of the household, gender, whether single or multiple households, and area (high or low endemic) were included in the model. The incidence rate ratios and their confidence intervals were computed.

#### Ethical considerations

Ethical clearance to conduct the study was obtained from the Ethics Committee of the Faculty of Medicine, University of Kelaniya, Sri Lanka and the Ethical Review Board at WHO, Geneva. Permission to conduct the study was obtained from the Ministry of Health and the Western Provincial Health Authority of Sri Lanka. Permission was also obtained from local government authorities, respective Medical Officers of Health, the Central Environment Authority and school principals.

Enrollment of households in the study was done after obtaining informed consent from heads of households. All households in the intervention area were provided with garbage separation bags, compost bins and plants free of charge. There was no restriction of any services or information to households of control clusters that was routinely available to the general public.

## Results

### Household characteristics in the intervention and control groups

803 households in the intervention group and 790 households in the control group were included in the study. There were no differences in the age and gender distributions of the heads of households and no differences in the number of family members per household between the treatment and control groups (Table 1). The heads of households of the intervention group were more educated, less frequently unemployed, owned a larger extent of land and had more housing and family units per household as compared to the control group. The differences were relatively small although statistically significant.

**Table 1:** Characteristics of households

Characteristic	Control group (n = 790)	Intervention group (n = 803)	Significance test (chi square test, p-value)
Age of head of household			
<50	443 (51.6%)	416 (48.4%)	2.164 (0.141)
≧50	347 (47.9%)	378 (52.1%)	
			
Gender of head of household			
Male	640 (48.9%)	672 (51.2%)	3.435 (0.064)
Female	150 (54.9%)	123 (45.1%)	
			
Education level of head of household			
<O Level	371 (53.7%)	320 (51.4%)	7.256 (0.007)
≧O Level	419 (46.97%)	475 (53.1%)	
			
Family units per household			
1	744 (51.7%)	696 (48.3%)	16.000 (<0.001)
>1	37 (20.4%)	144 (79.6%)	
			
Land ownership			
<10 perches	548 (53.7%)	472 (46.3%)	17.260 (<0.001)
≧10 perches	242 (42.8%)	323 (57.2%)	
			
Family members per household			
≤5	642 (50.8%0	621 (49.2%)	1.658 (0.198)
>5	158 (46.9%)	179 (55.1%0	
			
Employment status of head of household			
Employed	642 (49.7%)	649 (50.3%)	21.018 (<0.001)
Retired	26 (30.2%)	60 (21.018%)	
Unemployed	132 (59.2%)	91 (40.8%)	

### Results of the process analysis

Based on findings of the situational analysis (Phase I of the study) and discussions with stakeholders, it was decided to establish a solid waste management system at the local level to control dengue vector breeding in the most productive small discarded water containers. The system was developed with the support of local authorities and the Central Environment Authority with community ownership and participation. The project involved the distribution of low-cost compost bins free of charge among all households in the intervention area. There was regular intensified garbage collection from the local authority during the project period and beyond in the intervention clusters. In the control clusters, garbage collection was done as usual. The qualitative study revealed that the different partners worked in a coordinated way together complementing each other (see below on stakeholder analysis).

### Gender analysis

In the Focus Group Discussions, women were identified as the key actors in the entire process of cleaning homesteads and solid waste management at household level. Women spend more time at home than men, especially during the daytime. Culturally, the mother is the key figure guiding children in their day-to-day practices as well as in children’s educational process. Therefore, project activities centred around women as their role in the community enabled them to be better contributors to the waste management system.

### Results of the stakeholder analysis

All stakeholders involved in dengue vector management were identified through various sources, which included the local government authorities; the Medical Officer of Health of the area; religious leaders; public health inspectors; school principals and teachers; officers of the Central Environment Authority and other community leaders. A series of in-depth discussions were held and the strengths and weaknesses of stakeholders and gaps in effective waste management at the local level were identified and documented. 8–20 stakeholders participated in each meeting.

The analysis of roles, capacities and contributions of the stakeholders revealed the following:

1. The research team had the best technical expertise and capacity to coordinate all activities initially. The role was then passed on to the local health authorities before the completion of the project.
2. The primary stakeholders took the initiative in providing leadership and liaised with all stakeholders.
3. The secondary stakeholders assisted the primary stakeholders in providing necessary logistic support for solid waste collection with political commitment.
4. The volunteers and community provided support in the coordination of all activities.

### Community involvement

In each intervention cluster a ‘shramadana’ campaign, a voluntary effort to clean the outdoor environment, was conducted, eight in total. On average, over 100 persons participated in each campaign. Additionally, the participation of community members was crucial in all the other activities focusing on households.

### Entomological impact of the intervention

At baseline (round 1), there were no differences in any of the entomological indices (Table 2). Using the main outcome variable (pupae per 100 persons (PPP) index) a significant reduction of *Aedes* vectors 3 months (round2), 9 months (round 3) and 15 months after start of the intervention package was determined (Fig. 2). As there were differences at baseline between intervention and control groups, the reduction in the entomological indices as a percent of the baseline values was analyzed at cluster level (Table 2). The difference in the percent reduction of pupae per 100 persons from baseline to final follow up between intervention and control group was of borderline significance (p = 0.067).

**Figure 2:**
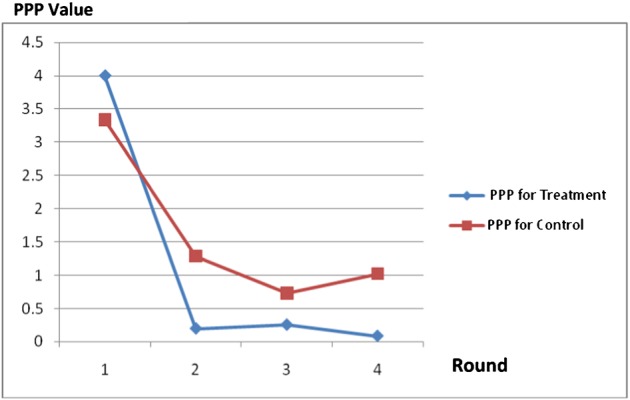
Comparison Pupa per 100 persons Index (PPP) for Control and Treatment Clusters.

**Table 2:** Percent reduction of pupae per 100 persons index adjusting for baseline differences

Index	Control group (mean reduction % ±sd)	Intervention group (mean reduction %±sd)	Significance between intervention and control groups (p-value)
Pupae per 100 persons			
Round 2 vs round 1	−0.667±0.01	−0.935±0.01	0.275
Round 3 vs round 1	−0.826±0.01	−0.940±0.01	0.547
Round 4 vs round 1	−0.716±0.01	−0.982±0.01	0.067

When modeling the number of pupae at household level adjusting for the baseline number of pupae and other factors (such as gender and education of head of household, number of housing units and endemicity, which were significantly different at baseline between treatment and control groups using the negative binomial model), the number of pupae in the treatment areas was significantly lower than that of the control areas in the three follow-up surveys. Only in rounds 2 (3 months) and 3 (9 months after start of the intervention), the number of pupae was higher in houses with less educated heads of household (Tables 3 and 4).

**Table 3:** Summary statistics of a negative binomial model using the number of pupae at household level in round 2 as the response variable after adjusting for the baseline number of pupae

Variables	Model coefficient	SD	Incidence Rate Ratio(95% CI)
Intercept	−3.320	0.6892	
Treatment^1^	−1.721	0.4217	0.179 (0.078–0.409)
Education status of head of household (<O/Level)^2^	0.914	0.3088	2.494 (1.362–4.568)
Male^3^	0.432	0.4220	0.036 (0.009–0.140)
Single housing unit^4^	−0.019	0.5497	0.981 (0.334–2.881)
Low endemicity^5^	−1.276	0.3486	0.279 (0.141–0.553)

^1^ Reference group is control group.

^2^ Reference group is heads of households with an education above O Level.

^3^ Reference group is female gender.

^4^ Reference group is more than one housing unit.

^5^ Reference group is areas with high endemicity.

**Table 4:** Summary statistics of negative binomial model using the number of pupae at household level in round 3 as the response variable after adjusting for the baseline number of pupae

Variables	Model coefficient	SD	Incidence Rate Ratio (95% CI)
Intercept	−2.225	0.7418	
Treatment^1^	−1.081	0.4050	0.339 (0.153–0.750)
Education status of head of household (<O/Level)^2^	−0.988	0.4177	0.368 (0.162–0.835)
Male^3^	−0.380	0.4422	0.688 (0.287–1.626)
Single housing unit^4^	0.022	0.6309	1.022 (0.297–3.520)
Low endemicity^5^	−1.172	0.4111	0.310 (0.138–0.694)

^1^ Reference group is control group.

^2^ Reference group is heads of households with an education above O Level.

^3^ Reference group is female gender.

^4^ Reference group is more than one housing unit.

^5^ Reference group is areas with high endemicity.

#### Larval indices before and after intervention

Larval indices reflect the presence or absence of vectors but not vector densities.3,^4^ In our study there were no differences in HI and CI between the two groups in any of the rounds. The BI in the intervention group was significantly lower than the control group after 15 months but not in rounds in between. The larval indices at the end of the study period were significantly lower than those at baseline though no statistically significant differences between intervention and control arms could be determined except for the Breteau index. The mean Breteau index dropped from 11.75 and 9.75 at baseline to 3.13 and 6.25 in survey round 4 in the intervention and the control clusters, respectively.

## Discussion

The presence of solid waste around households, such as cans, car parts, bottles, old and used tyres, plastic materials, broken clay, glass vessels and coconut shells, created outdoor breeding sites for *Aedes* mosquitoes and represented in our ecosystem the most productive container types. Maintaining solid waste for a long time often in excess of seven days supports the breeding of *Aedes aegypti*^1^ and increases the transmission of dengue. If the frequency of collection and disposal of solid waste increases, it should theoretically control *Aedes* breeding and, thus, reduce dengue transmission.

The effectiveness of a community-based intervention aimed at reducing *Aedes* breeding in the community as a strategy for reducing dengue transmission was evaluated in this study although it was not possible to exclude control neighborhoods (clusters) from all project activities. An intervention of proper solid waste management at household level was selected based on entomological assessments made during the situational analysis of Phase 1 of this project where the most important breeding habitats of *Aedes* mosquitoes in this community were discarded containers. The intervention comprised promotion of composting biodegradable household waste, raising awareness on the importance of solid waste management in dengue control and improving garbage collection with the assistance of local government authorities. The hypothesis that we postulated at the outset was that the introduction of a proper solid waste management programme will thus reduce *Aedes* breeding in the community.

There was a significant reduction in the major outcome variable (number of pupae per 100 persons, PPP) between intervention and control arms at the end of the study after adjusting for baseline values and other characteristics that differed between the intervention and control groups at baseline. *Aedes* pupae per 100 persons index is considered a direct proxy for adult *Aedes* mosquitoes densities. The pupal density is a better index of adult *Aedes* mosquitoes as the development of larvae to pupae is dependent on a number of environmental and human behavioral factors such as rainfall patterns, temperature, garbage disposal and collection practices. The large differences between the number of larvae and number of pupae found in both groups of clusters further strengthen the argument that pupae are a better proxy for adult *Aedes* mosquitoes as compared to larvae.

Of all the households surveyed in both the intervention and control clusters, there were only a few households that had *Aedes* larvae and pupae. Despite this observation, there was reduction in the number of *Aedes* larvae and pupae per household in both the intervention and control clusters, though the reduction was greater in households in the intervention clusters. The reduction in the larval and pupal densities in the control clusters may be due to behavioral changes that may have taken place during the project or due to other reasons such as reduced rainfall during the follow-up studies. The finding that only a few households harbored *Aedes* pupae has implications for dengue control programmes as it is possible to better target interventions to these populations and their productive container types.

Urbanization is a major problem faced by developing countries that has imposed a severe strain on public health services including provision of solid waste management services. In Sri Lanka, 60% of solid waste at household level is bio-degradable; this organic fraction makes a relatively large contribution to the total weight due to its high density and water content.^9^ Based on these findings, the composting programme was initiated in the Gampaha district of Sri Lanka. The district comprises urban, suburban and rural areas and is typical of similar areas in other parts of the country. Hence, the findings of this study are applicable to other areas where similar interventions may be applied.

Even though we have demonstrated a reduction in the number of *Aedes* pupae in the intervention clusters, the ultimate goal is to show a reduction in the number of dengue cases. The number of dengue cases reported during the study period was low in both intervention and control clusters; however, when looking at the surrounding areas of our study clusters, the number of reported dengue cases was lower in the wider areas around the intervention clusters compared to the areas in which the control clusters were located. Although the intervention was focusing on only two neighborhoods with 400 houses, the community-based solid waste management intervention was also beneficial for the surrounding communities who received a more frequent and better organized garbage collection.

The intervention was designed and implemented taking into account the results of the situational analysis done in Phase I of the study, which included targeting females to be the most receptive population and taking into consideration the capacities and potential roles of all stakeholders. The mobilization of the community was essential for the successful implementation and sustainability of the programmes. In designing the intervention, emphasis was laid on establishing volunteer groups that are continuing with the programme and targeting school children to ensure better practices among the next generation. Volunteers organized successful cleaning campaigns and liaised well with local authorities in ensuring a regular garbage collection system. The challenge is to ensure the sustainability of the intervention without the inputs of the research team; this we propose to assess in due course.
